# Maternal feeding behaviour and young children's dietary quality: A cross-sectional study of socially disadvantaged mothers of two-year old children using the Theory of Planned Behaviour

**DOI:** 10.1186/1479-5868-8-65

**Published:** 2011-06-23

**Authors:** Vivien Swanson, Kevin G Power, Iain K Crombie, Linda Irvine, Kirsty Kiezebrink, Wendy Wrieden, Peter W Slane

**Affiliations:** 1 Department of Psychology, University of Stirling, Stirling, FK9 4LA, UK; 2 Department of Clinical Psychology, NHS Tayside, 7, Dudhope Terrace, Dundee, DD3 6HG, UK; 3 Department of Public Health, University of Dundee, Mackenzie Building, Kirsty Semple Way, Dundee DD2 4BF, UK; 4 Health Services Research Unit, University of Aberdeen, Foresterhill, Aberdeen, AB25 2ZD, UK; 5 School of Pharmacy and Life Sciences, The Robert Gordon University, Schoolhill, Aberdeen, AB10 1FR, UK; 6 Erskine Practice, Arthurstone Medical Practice, Dundee, DD4 6QY, UK

## Abstract

**Background:**

Having breakfast, eating food 'cooked from scratch' and eating together as a family have health and psychosocial benefits for young children. This study investigates how these parentally determined behaviours relate to children's dietary quality and uses a psychological model, the Theory of Planned Behaviour (TPB), to investigate socio-cognitive predictors of these behaviours in socially disadvantaged mothers of young children in Scotland.

**Method:**

Three hundred mothers of children aged 2 years (from 372 invited to participate, 81% response rate), recruited via General Practitioners, took part in home-based semi-structured interviews in a cross-sectional survey of maternal psychological factors related to their children's dietary quality. Regression analyses examined statistical predictors of maternal intentions and feeding behaviours.

**Results:**

Mothers of children with poorer quality diets were less likely than others to provide breakfast every day, cook from 'scratch' and provide 'proper sit-down meals'. TPB socio-cognitive factors (intentions, perceived behavioural control) significantly predicted these three behaviours, and attitudes, norms, and perceived behavioural control significantly predicted mothers' intentions, with medium to large effect sizes.

**Conclusions:**

Interventions to improve young children's dietary health could benefit from a focus on modifying maternal motivations and attitudes in attempts to improve feeding behaviours.

## Background

Living in a socially disadvantaged family is linked with poorer quality diets and negative health outcomes for young children, including obesity. Poorer knowledge of healthy eating, more negative attitudes towards food and eating, fewer financial and practical resources (e.g. food supply, cooking facilities), lower maternal self-confidence and fewer food preparation skills are all associated with less healthy eating behaviours [1,2]. Issues of cost and access to high quality food are also crucial. Additionally, the fragmented living environment of some people in areas of social disadvantage may not be conducive to providing traditional family meals [2,3]. For example some families may not own a dining table, irregular lifestyles or shift working may make regular mealtimes difficult, and single parents may lack social or community support in relation to childcare. A recent Scottish report suggested that 32% of children aged between 2 years 10 months and 4 years 10 months ate in the living room rather than the kitchen/dining room, suggesting a less structured approach to mealtimes. This rate doubled for younger teenage mothers and those in the lowest income category [3]. Disadvantaged parents are generally a 'hard to reach' group for researchers, and there are few studies which aim to understand how their attitudes and motivations towards food and eating behaviours influence their children's diet. We aimed to investigate this in the current study.

Parents (most frequently mothers) generally have primary responsibility for determining what, where, when and how their children eat [4,5] and dietary behaviours established in childhood continue into adulthood [6]. One study suggested that eating behaviours in children are established by the age of 4, and generally persist thereafter [7]. Parental influence is therefore greatest in the early years, and there is potential for using psychosocial interventions to influence parents' food preparation behaviours during this period [8]. Provision of children's food has a symbolic meaning in the context of parenting and family relationships. For example, preparing 'home-made' food or giving children breakfast may be perceived as a quality of a 'good mother' [9]. Eating together 'as a family' may also foster better family relationships and better attitudes to food, for example by addressing food neophobia in young children and modelling 'healthy' or socially desirable eating behaviours. However psychological factors, such as attitudes to food preparation and parental self-efficacy regarding food provision are much less researched than energy balance factors. The current study aimed to investigate socio-cognitive predictors of maternal feeding behaviour and their relationship with young children's dietary quality using the Theory of Planned Behaviour (TPB) [10], as part of a larger study into mothers' dietary knowledge and their attitudes to their children's diet [11]. The TPB is an explanatory motivational model which indicates how attitudes, social norms and perceived behavioural control (PBC) combine to explain behavioural intention, and how intentions and PBC directly predict behaviour. It is a widely used social cognition model and is clearly specified and reliable. The TPB has frequently been applied to dietary behaviours. A recent review [12] identified 19 dietary studies focusing on different behaviours including healthy eating, reducing fat intake, increasing fruit and vegetable intake, restricting sugar intake and choice of breakfast foods. Approximately 41% of variance in intention and 16% of variance in eating behaviour was statistically predicted in these studies.

### Identifying relevant behaviours for the current study

A qualitative pilot study carried out in two socially deprived areas in Scotland included 30 mothers of young children aged two years, in 6 focus group sessions. The data were subjected to a systematic thematic analysis [13] which included reading, re-reading, annotating and charting themes and sub-themes. Themes were considered valid where they had been mentioned by several participants. Three overarching themes emerged: mothers' concern for their child's dietary health; control over children's eating behaviours; and the family environment. This latter theme identified aspects of family life which mothers perceived as affecting children's dietary quality and factors influencing mothers' motivation regarding their child's dietary health. (Further details of the focus group methods and analysis are available from the authors on request). Several food-related health behaviours were identified, including restricting the child's diet and introducing novel foods. However, the most frequently mentioned were providing breakfast, eating together as a family and cooking 'proper meals' from 'scratch' (assembling ingredients). We therefore focused on these behaviours.

### Eating Breakfast

A review of 47 studies showed that eating breakfast has a critical role in determining child health, although breakfast consumption has declined in all age groups in western cultures [14]. Breakfast is an important meal since it sets energy levels and is associated with better cognitive functioning and academic performance, with improved mood effects for school-aged children [15]. Eating breakfast is particularly important for children at 'nutritional risk' including those from socially disadvantaged backgrounds [16]. The role of parents is pivotal for including breakfast as part of young children's morning routine. Maternal self-efficacy (perceived control) may be important, but has not previously been investigated in relation to pre-school children.

### Family Mealtimes

The frequency of 'family' mealtimes where children and adults eat together also has an important impact on aspects of child development. There are both nutritional and psychosocial benefits from family mealtimes. Children in families eating together have healthier diets [17], eat more fruit and vegetables and novel foods and less fried food and sugary drinks [6,18]. Benefits also occur for young children in relation to behavioural modelling of food consumption, socialisation and language development, literacy skills and addressing behavioural problems [19]. Family mealtimes are linked with less television viewing, which is also related to healthier eating [20].

### Cooking from scratch

'Cooking from scratch', (being able to assemble a meal from basic ingredients) as opposed to eating pre-prepared or take-away food, is related to empowerment and improved nutrition [21]. Ability and/or willingness to 'cook from scratch' in younger adults has recently declined, perhaps a reflection of changing women's roles, including working longer hours outside the home, changes in eating habits, and general loss of culinary skills [22].

The current study aimed to investigate which socio-cognitive determinants as specified in the TPB would statistically predict maternal feeding motivations (intentions) to carry out these behaviours (providing breakfast, preparing meals from scratch and eating meals together as a family). The study also aimed to investigate how these maternal behaviours were related to young children's dietary quality.

## Methods

We carried out a cross-sectional questionnaire survey of a cohort of socially disadvantaged mothers of young children aged 2 years [11]. The questionnaire was presented to mothers in their homes using a laptop computer, with a user-friendly format using pictures and illustrative graphics. Detailed questions on dietary quality were included, based on national dietary guidelines [23-25] and recommendations from the 'EatWell Plate'[26], the updated version of the 'Balance of Good Health' (BOGH). This is a pictorial representation of the five food groups: including fruit and vegetables; meat, fish, eggs and beans; bread, rice, potatoes, pasta; milk and dairy; and fats and sugars, indicating the proportion of the diet that should be provided from each group.

### Participants

In this study we defined social disadvantage by area of residence, using postcode data. Participants were identified from families registered with general practitioners (GP) in areas of high social deprivation, using the Scottish Index of Multiple Deprivation (SIMD) [27]. Ten GP practices were selected from the two most deprived deciles in two Scottish NHS Health Board areas, and anonymised lists of children aged 2 years were provided, from which children living in areas of high deprivation (low SIMD scores) were randomly selected. The study was exploratory, and we were not able to test hypotheses about expected effect sizes. The sample size was therefore determined using 95% confidence intervals, with α 0.05, and a specified target of 300 women. In the absence of variance estimates we set a maximum 95% confidence interval for a binary variable at ± 5%, assuming maximum variance (p = 50%). This would allow us to calculate effect sizes with a high degree of precision for future work. Anticipating an approximately 25% refusal/drop-out rate, mothers of 372 children were invited to take part using GP generated letters sent to their home address. Particular efforts were made to obtain a sample of mothers representing socially disadvantaged SIMD categories. Out of 372 invited, 49 refused, 18 were not contactable and 5 did not fit inclusion criteria (81% response rate). Families where the mother was not the main caregiver were excluded as they may differ significantly in their behaviours, beliefs and attitudes to food. Three hundred mothers of two year old children took part. Their ages ranged from 18 - 34 (mean 24.9, SD3.2). Many were single-parents or had no current partner (35%, n = 105), and 43% (128) were the only adult in the household. Most (91%, n = 272) were unemployed, the remainder worked part-time. Most (81%, n = 243) lived in rented social housing. The index children were 49% (n = 148) female, 27%, (n = 82) had no siblings, over half (57%, n = 171) had older siblings, and 27%, (n = 81) had younger siblings. Children's mean age was 30.3 (SD3.2) months, range 24-36 months. Recruitment details are described in detail elsewhere [28]. Ethical approval was given by the local NHS NRES Research Ethics Committee.

### Measures

The dietary quality score was based on the Balance of Good Health (BOGH) plate (now called the EatWell plate [26]. This shows the balance of food groups recommended for a healthy diet, roughly one third fruit and vegetables, one third starchy carbohydrate and the other third made up of milk and dairy products (15%), meat, fish and alternatives (12%) and a small proportion of fatty and sugary foods (8%). We used dietary questions from previously used questionnaires to determine the frequency of the child's daily consumption of food in these categories, estimated over the past month [29-31]. Our initial aim was to measure how many children achieved a balanced diet as recommended in the EatWell plate (i.e. a ratio of 3:3:1:1:1 for fruit and vegetables: starchy carbohydrates: dairy: meat: fats/sugar). However, evidence suggests few children achieve recommended levels on all food groups [32] and this was borne out in our initial analysis, which showed there were no children in our sample whose diet matched these criteria.

We therefore developed criteria which allowed more flexibility, being more representative of the diet of 2-3 year old children [5]. This allowed for a higher proportion of fats and sugary foods. Table 1 indicates the proportion of children who were classified as having a better quality diet in each food group. To investigate the relationship between dietary quality and behaviours, children were first allocated a dichotomous score according to whether or not they achieved criteria in all five 'Balance of Good Health' food groups (BOGH1). Second we investigated more lenient criteria based on achieving a good diet in four out of five food groups (BOGH2).

**Table 1:** Number and percentage of participants achieving Balance of Good Health (BOGH) criteria

*BOGH Criterion*	Achieved n =	%	*Notes*
Eats 2 or more portions of bread, other cereals and potatoes daily	*203*	*68*	*Includes breakfast cereals, rice and pasta*

Eats 2 or more portions of fruit or vegetables daily	*184*	*61*	*Includes all types (frozen, fresh), excluding pulses (see below). Fruit juice can only contribute one portion a day maximum*.

Eats one or more portions of dairy products daily	*300*	*100*	*All milk including cow, goat, soya, etc*

Eats one or more portions of meat, fish or alternatives daily	*272*	*91*	*Includes both processed and non-processed food. Includes pulses*

Eats no more than two high fat or high sugar snacks daily	*104*	*34*	*Includes sweets, chocolate, crisps, savoury snacks*

Achieves all five of above	*45*	*15*	

Achieves four of above	*70*	*23*	

### Theory of Planned Behaviour

TPB items were scored using 5 point likert scales, with anchors 'strongly disagree' (1) to 'strongly agree' (5) for 'recommended' frequency of behaviour, intention, subjective norm and perceived behavioural control, and 'not at all important' (1) to 'very important' (5), and 'very unenjoyable'(1) to 'very enjoyable'(5) for attitudes. Higher values therefore represented more positive scores.

### Behaviour

Three salient maternal feeding behaviours were selected according to importance from the focus groups, being related to dietary quality in previous literature, with specific health benefits but underpinned by different psychosocial and practical skills. The behaviours were: a) Provide breakfast for my child, b) Cook meals from 'scratch' (i.e. assembled from ingredients), c) Provide a 'proper sit down meal'. We note that there could be ambiguity around the use of the term 'proper'. In some social groups it could imply socially desirable nutritional and psychosocial aspects of family mealtimes - contrasting proper (good) with improper (bad) behaviours. Revisiting data from the focus groups we noted that this term was commonly used with an alternative meaning by our participants, as a label to differentiate 'formal' from 'informal' mealtimes (for example, eating in front of the television would be judged to be informal). We have therefore retained this label in the text as representing 'family' meals eaten together since we felt it was important to use the participants' own language in constructing and administering the questionnaire.

Behaviours were measured in two ways:

a) Direct questions on actual frequency of the behaviour in the past week, e.g. 'thinking about the last week, how often did (child) have breakfast?' (score range 0-7),

b) Indirect questions based on 'recommended' frequency of behaviours from guidelines and expert opinion: 'do you give (child) breakfast *every day*?'; 'do you cook meals from scratch *at least four times a week'*, and 'do you provide a proper sit down meal *at least once a week'*, (all scored from 1, strongly disagree to 5 strongly agree).

Providing breakfast does not generally require food preparation skills but necessitates maintaining a routine in day to day life. Having a 'proper sit-down' meal was defined as eating as a family, and sitting round a table, rather than in front of the TV or on one's own. This draws on maternal food preparation skills and allows adults to model social and communication skills. 'Cooking from scratch' requires sourcing and preparation of basic ingredients, and time management. The interviewer explained this meant assembling a meal from ingredients, including the use of pre-prepared ingredients such as pasta sauces. To reduce priming and social desirability bias in responding, the behaviour questions were asked *before *the other TPB components (attitudes, norms, control and intentions) to make sure participants were clear about the definitions of each.

### Intention

We measured intentions using two questions representing 'planning' and 'wanting' for each of the behaviours [12]. For analysis, a combined intention variable was calculated using the mean of the two questions. Cronbach's alpha values were α = .72 for 'intention to provide breakfast' (mean 7.06, SD 1.7); α = .73 for 'cooking from scratch' (mean 6.62, SD 1.57), and α = .80 for having a 'sit-down' meal, (mean 7.48, SD 1. 9).

### Attitudes

Attitudes were measured using one instrumental (cognitive) and experiential (affective) evaluative statement for each behaviour [12,33,34], e.g. 'giving (my child) breakfast every day would be ...' unimportant/important, or unenjoyable/enjoyable.

### Subjective Norm

The subjective norm reflects perceived social pressure to perform a behaviour. Subjective norms include 'descriptive' (perceptions of what other people do) and 'injunctive' norms (what others think you should do) [35] which we measured for each behaviour. For example; 'most people give their child a proper sit-down meal at least once a week'; and 'most people think I should give (my child) a proper sit-down meal at least once a week'.

### Perceived Behavioural Control

Perceived behavioural control reflects the belief that one can carry out a behaviour to achieve particular outcomes. Ajzen [34] recommends assessing perceived self-efficacy and perceived controllability. Conner and Sparks (2005, pp189-192) [12] provide a useful summary of measurement issues. Controllability and self-efficacy were assessed in this study: e.g.' Whether or not I cook a meal from scratch for (child) at least 4 times a week is entirely up to me' (controllability), and, 'I am confident that I can cook a meal from scratch for (child) at least 4 times a week' (self-efficacy).

### Other Health Behaviours

All participants in the study were resident in areas of social disadvantage as defined by postcode [27]. However we know that some health behaviours can compound future social disadvantage for young children via their impact on health [6,25]. As additional markers of social disadvantage, participants were asked whether they smoked (yes/no) and if their child had been breastfed (yes/no). They were also asked about their child's television viewing (number of hours per day) and whether their child played outside (yes/no).

### Analysis

Analysis was carried out using SPSS 16 statistical package [36]. For the BOGH score, we created dichotomous variables which allocated children to better quality/ poorer quality dietary groups, a) if they achieved our criteria on all food groups (BOGH1), and b) to achieve more power in the analysis we also summed the number of criteria achieved and dichotomised the sample on the basis of meeting all but one of the criteria (BOGH2). Descriptive statistics were used to check normality of variables used in the analysis. The direct measure of frequency of breakfast was highly negatively skewed, (skewness = - 3.6) so we used a log transformation of this variable (reducing skewness to 2.0) in correlation and regression analysis. All other variables were found to be within acceptable parameters. We examined intercorrelations of direct and indirect behaviours using Pearson r. Since group sizes were unequal, with unequal variance in some cases, non-parametric statistics (Mann-Whitney U) were used to assess group comparisons. As in other TPB studies [12] TPB variables were treated as interval data in the analyses. TPB models were tested using multiple regression analysis (entering variables in one block) with intention and actual and recommended measures of behaviour as outcomes.

## Results

Two thirds of participants smoked, (60%, n = 181) and only 22% (n = 66) had breastfed their child. About half (45%, n = 138) of the children went to nursery during the day. Some children received food at nursery, including morning snack (24%, n = 72), lunch (10%, n = 31) and afternoon snack (3%, n = 8). Most (82%, n = 245) children watched television for over 4 hours per day, only a fifth (22%, n = 67) ever played outside and a third of these only did so at nursery.

### Dietary Quality

Only a small proportion of children could be considered to have a balanced healthy diet using the 'EatWell Plate'[26] (the new version of BOGH). Although all or most participants achieved some of the criteria as shown in Table 1, only 15% (n = 45) achieved all five (BOGH1), and 23% (n = 70) achieved four out of five (BOGH2). All participants reported their child ate one or more portions of dairy products daily. Most ate one or more portions of meat or fish. Two thirds ate two or more portions of bread/cereals, and two or more portions of fruit/vegetables daily. Two thirds of children ate more than two high fat or high sugar snacks daily.

### Maternal Feeding Behaviours

Mean values for actual frequency of behaviours showed that providing breakfast was more frequent in the previous week than cooking from scratch and having a proper sit down meal as shown in Table 2.

**Table 2:** Means, standard deviations and correlations for actual frequency and recommended frequency behaviour and TPB variables for 'Providing Breakfast', 'Cooking from Scratch', and 'Having a Proper Sit-down meal'

Variable												
**Breakfast**		**Mean**	**SD**	**1a**	**1b**	**2**	**3**	**4**	**5**	**6**	**7**	**8**

**1a#**	BEH-Freq / log freq	6.46/ .11	1.32/.21									

**1b**	BEH-Rec	3.62	.96	.35	1.0							

**2**	INT	7.06	1.67	.27	.70	1.0						

**3**	Att-I	3.93	.66	.10^b^	.60	.67	1.00					

**4**	Att-E	3.04	.47	.28	.30	.37	.30	1.00				

**5**	SN-I	3.20	1.0	.^.^12*	.27	.38	.43	.22	1.00			

**6**	SN-D	3.35	.88	.07 ^b^	.31	.32	.39	.20	.36	1.00		

**7**	PBC-C	3.96	.69	.28	.31	.35	.16**	.28	-.07^b^	-.05 ^b^	1.00	

**8**	PBC-SE	4.12	.58	.03^b^	.36	.48	.29	.31	.06 ^b^	.20	.52	1.00

**Cooking from Scratch**		**Mean**	**SD**	**1a**	**1b**	**2**	**3**	**4**	**5**	**6**	**7**	**8**

**1a**	BEH-Freq	3.25	1.9	1.0.								

**1b**	BEH- Rec	2.99	1.04	.61	1.00							

**2**	INT	6.62	1.57	.80	.62	1.00						

**3**	Att-I	3.34	1.00	.73	.50	.77	1.00					

**4**	Att-E	2.59	.93	.55	.41	.62	.66	1.00				

**5**	SN-I	2.63	.94	.53	.30	.54	.58	.41	1.00			

**6**	SN-D	2.16	.90	.37	.43	.39	.36	.23	.29	1.00		

**7**	PBC-C	3.49	.86	.32	.28	.32	.24	.16**	.13*	.15**	1.00	

**8**	PBC-SE	3.45	.95	.36	.41	.29	.24	.15**	.12*	.12*	.52	1.00

**Sit-down meal**		**Mean**	**SD**	**1a**	**1b**	**2**	**3**	**4**	**5**	**6**	**7**	**8**

**1a**	BEH-Freq	2.48	1.20	1.0								

**1b**	BEH-Rec	4.07	1.15	.77	1.0							

**2**	INT	7.48	1.92	.79	.84	1.0						

**3**	Att-I	3.76	1.03	.71	.56	.66	1.0					

**4**	Att-E	3.09	1.24	.72	.44	.57	.62	1.0				

**5**	SN-I	2.82	1.08	.16**	.40	.45	.33	.30	1.0			

**6**	SN-D	2.10	.90	.37	.26	.24	.18**	.13*	.14*	1.0		

**7**	PBC-C	3.55	.91	.28	.36	.36	.26	.16**	.19**	.03 ^b^	1.0	

**8**	PBC-SE	3.87	.86	.47	.61	.54	.31	.25	.21	.12*	.46	1.0

# Correlations presented are using log of this variable (actual frequency of breakfast)

All correlations > .20 significant at p < 0.001; ** = p < 0.01; * = p < 0.05; ^b^**not significantly correlated**

Key: BEH -Freq: Actual frequency of behaviour; BEH-Rec: Recommended frequency of behaviour; Intention: Combined Wanting/Planning; AttI: Attitude-Instrumental; Att-E: Attitude Experiential; SN-I: Subjective norm-Injunctive; SN-D: Subjective norm-descriptive; PBC-C: Perceived Behavioural Control-Controllability; PBC-SE: Perceived behavioural control-Self-efficacy.

The pattern for 'recommended' frequency (indirect measure) of behaviours was slightly different. Participants were more likely to agree they had a sit-down meal than other behaviours. There was a strong association between actual and recommended frequency of each behaviour as shown in Table 2 (all p < 0.001). Correlations between actual frequencies of breakfast with cooking from scratch (r = .16, p = 007) and eating a proper sit down meal (r = .17, p = .003) were small, whereas frequency of cooking from scratch and having a sit-down meal were highly correlated (r = .74, p <.0001) as might be expected. Intercorrelations between the three recommended behaviour variables were modest (Pearson r = .24 to r = .26) suggesting they were related but conceptually distinct constructs.

### Maternal Feeding Behaviours and Dietary Quality

Non-parametric statistics (Mann-Whitney U) were used to compare direct and indirect behaviours in relation to dietary quality, a) comparing those who met all criteria (n = 45) with those who did not (n = 255) (BOGH1) and b) comparing those who met four out of five criteria (n = 70, with those who did not (n = 230), (BOGH2) as shown in Table 3.

**Table 3:** Dietary quality and eating behaviours: (a) actual frequency and b) recommended frequency for participants whose children had better and poorer quality diets, comparing (BOGH1)^1 ^and (BOGH2)^2 ^criteria

Feeding behaviour	Mean (SD)	Mean (SD)	Mann-whitney U (Z-Scores), P
***a) Actual frequency (times per week)***	**BOGH1: Better** **quality diet** **(n = 45, 15%)**	**BOGH1: Poorer** **quality diet** **(n = 255, 85%)**	

**Preparing Breakfast**	6.91 (.36)	6.4 (1.4)	-2.86, p = .004

**Cooking from Scratch**	3.27 (1.9)	3.16 (1.8)	-.25, p = .79

**Sit-down meal**	3.6 (2.4)	2.5 (2.6)	-2.75, p = .006

	**BOGH2: Better quality diet** **(n = 70, 23%)**	**BOGH2: Poorer** **quality diet** **(n = 230, 77%)**	

**Preparing Breakfast**	6.9 (.42)	6.3 (1.5)	-3.85, p = .001

**Cooking from Scratch**	3.9 (1.8)	3.0 (1.9)	-3.07, p = .002

**Sit-down meal**	3.7 (2.5)	2.4 (2.5)	-3.95, p <.0001

**b) Recommended frequency^3^**	**BOGH1: Better quality diet** **(n = 45, 15%)**	**BOGH1: Poorer quality diet** **(n = 255, 85%)**	

**Preparing Breakfast**	3.81 (.79)	3.65 (1.1)	-1.85, p = .06

**Cooking from Scratch**	3.16 (1.0)	2.90 (1.0)	-1.9, p = .23

**Sit-down meal**	4.65 (.77)	3.97 (1.2)	-3.98, p <.0001

	**BOGH2: Better quality diet** **(n = 70, 23%)**	**BOGH2: Poorer quality diet** **(n = 230, 77%)**	

**Preparing Breakfast**	3.95 (.72)	3.52 (1.01)	-3.01, p = .002

**Cooking from Scratch**	3.24 (1.05)	2.91 (1.02)	-2.96, p = .021

**Sit-down meal**	4.43(.98)	3.96(1.18)	-4.48, p <.0001

^1^BOGH1: Achieves all 5 'Balance of Good Health' criteria (see Table 1)

^2^BOGH2: Achieves 4 out of 5 'Balance of Good Health' criteria

^3^Preparing breakfast every day; Cooking from scratch more than four times per week; Proper sit-down meal at least once a week.

The actual frequencies of preparing breakfast and having a proper sit-down meal were significantly greater for mothers of children with better quality diets using both BOGH1 and BOGH2 criteria (respectively p = .004; p = .006 for BOGH1 and p <.001 and p <.001 for BOGH2 ). There was no significant difference for 'cooking from scratch' for BOGH1, but this became significant (p = .002) using more lenient BOGH2 criteria.

Examining the frequency of recommended behaviours showed a similar pattern. However, only having a proper sit-down meal was significantly more frequent for those with a better quality diet using BOGH1 criteria, whereas all three behaviours showed statistically significant differences according to dietary quality using more lenient BOGH2 criteria (respectively p = .002, p = .021, p <.001).

### Theory of Planned Behaviour Analysis

Correlations, means and standard deviations for the three behaviours (using direct (actual frequency) and indirect (recommended frequency measures) and TPB components are shown in Table 2. Most correlations between behaviours and TPB components were statistically significant. In general, subjective norms had higher values for providing breakfast than cooking from scratch and having a sit-down meal, suggesting this behaviour is seen as more socially desirable.

A series of linear regression analyses were carried out with behaviours (actual frequency and recommended frequency) as outcome variables to test the TPB model, as shown in Table 4. Despite some evidence of collinearity, diagnostics for the separate regressions showed no tolerance values less than 0.1, and average variance inflation factor (VIF) of around 1.5 which does not give cause for concern [37].

**Table 4:** Regression analyses predicting mothers' a) actual frequency and b) recommended frequency of behaviour for 'Providing breakfast', 'Cooking from Scratch' and 'Having a proper 'sit-down' meal'

*Breakfast^1^*	*B*	*SE B*	*Β*	*95% CI for B*	p =
***a) Actual frequency (log)***					

**INT**	-.04	.01	-.32	-.06 to -.03	.0001

**PBC-C**	-.11	.02	-.35	-.15 to -.07	.0001

**PBC-SE**	.14	.03	.36	.09 to .19	.0001

Model Adj R^2 ^= .19, F(3,296) = 23.65, p < 0.0001					

***b) Recommended frequency***					

**INT**	.39	.03	.68	.34 to .45	.0001

**PBC-C**	.12	.07	.09	-.01 to .26	.07

**PBC- SE**	-.04	.09	-.02	-.21 to .13	.65

Model Adj R^2 ^= .49, F(3,296) = 96.30, p < 0.0001					

***Cooking from scratch*** ***a) Actual frequency***	***B***	***SE B***	***Β***	***95% CI for B***	**p =**

**INT**	.93	.04	.76	.85 to 1.03	.0001

**PBC-C**	.02	.09	.01	-.15 to .20	.83

**PBC-SE**	.27	.08	-.13	.11 to .43	0.001

Model Adj R^2 ^= .66, F(3,296) = 193.63, p < 0.0001					

***b) Recommended frequency***					

***Int***	.37	.03	.55	.31 to .43	.0001

***PBC-C***	-.05	.06	-.04	-.17 to .07	.41

***PBC- SE***	.30	.06	.27	.31 to .43	.0001

Model Adj R^2 ^= .44, F(3,296) = 78.2, p < 0.0001					

***Sit down meal***	***B***	***SE B***	***Β***	***95% CI for B***	**p =**

***a) Actual frequency***					

**INT**	1.03	.06	.76	.92 to 1.15	.0001

**PBC-C**	-.05	.16	.02	-.28 to -.18	.64

**PBC-SE**	.20	.14	.07	.07 to .47	.15

Model Adj R^2 ^= .62, F(3,296) = 161.5, p > 0.0001					

***b) Recommended frequency***					

**Int**	.44	.02	.73	.40 to .48	.0001

**PBC-C**	.01	.04	.01	-.07 to .09	.81

**PBC- SE**	.27	.05	.20	.17 to .37	.0001

Model Adj R^2 ^= .74, F(3,296) = 283.4, p > 0.0001					

^1^Transformation (log) reduced overall skewness in the 'breakfast' actual frequency variable, but since the transformed variable remained skewed we also dichotomised this into 2 categories, 'every day' and 'less than every day', and carried out a logistic regression using the same predictors as a check. The model was significant, Chi Square = 45.71, p < 0.0001; Nagelkerke R^2 ^= .22, with all predictors being highly significant using the Wald test (all p < 0.0001).

TPB models tested for each behaviour were highly significant, and the percentage of variance predicted suggested sizeable effects for actual frequency and recommended frequency variables (predicted variance ranged from 19% to 74%). For providing breakfast, and having a proper sit-down meal, a larger percentage of variance was predicted using the indirect measure of behaviour (adherence to recommended frequency) as the outcome, whereas for cooking from scratch, actual frequency predicted 66% of variance, compared with 44% predicted for the indirect measure. Intentions were consistently statistically significant predictors of behaviour for all six models, and may have had a suppressor effect on control variables.

Effect sizes were also large for statistically predicting intentions from TPB variables, explaining 57% of variance for providing breakfast, 65% for cooking from scratch and 64% for providing a sit-down meal. as shown in Table 5.

**Table 5:** Regression analyses predicting mothers' intentions for 'Providing breakfast', 'Cooking from Scratch' and 'Having a proper 'sit-down' meal'

*Breakfast*	*B*	*SE B*	*Β*	*95% CI for B*	p =
Att-I	1.26	.12	.49	1.03 to 1.49	.0001

Att-E	.28	.15	.08	-.02 to .57	.065

SN-I	.22	.07	.13	.08 to .37	.003

SN-D	.05	.08	.03	.-.11 to .21	.52

PBC-C	.34	.11	.14	.12 to .57	.003

PBC- SE	.66	.14	.23	.39 to .92	.0001

Model Adj R^2 ^= .57, F (6,293) = 66.61, p < 0.0001					

***Cooking from scratch***	**B**	**SE B**	**Β**	**95% CI for B**	**p =**

Att-I	.76	.08	.48	.59 to .92	.0001

Att-E	.33	.08	.20	.18 to .48	.0001

SN-I	.20	.07	.12	.07 to .34	.004

SN-D	.20	.06	.12	.07 to .33.	.002

PBC-C	.18	.07	.10	.04 to .33	.013

PBC- SE	.11	.07	.07	-.02 to .24	.102

Model Adj R^2 ^= .65, F (6,293) = 92.63, p < 0.0001					

***Sit-Down Meal***	**B**	**SE B**	**Β**	**95% CI for B**	**p =**

Att-I	.65	.09	.36	.49 to .82	.0001

Att-E	.30	.07	.20	.17 to .46	.0001

SN-I	.33	.07	.19	.20 to .46	.0001

SN-D	.18	.08	.08	.03 to .33	.02

PBC-C	.10	.08	.05	-.06 to .27	.22

PBC- SE	.71	.09	.32	.53 to .89	.0001

Model Adj R^2 ^= .64, F (6,293) = 87.97, p < 0.0001					

Attitudes were consistently statistically significant predictors. Subjective norm-descriptive was the only non-significant predictor of intentions to prepare breakfast. PBC-self-efficacy was the only non-significant predictor of intentions to cook from scratch, and PBC-control was the only non-significant predictor of intentions to have a sit-down meal.

## Discussion

Encouraging healthy eating for very young children is crucial for governments' attempts to improve population health. Unhealthy diets in early childhood perpetuate in later childhood and beyond, suggesting mothers of young children have a clear opportunity to influence their children's long-term health during their early years [3]. Participants in the current study were all living in socially deprived areas. Demographic data from this sample, (including many single parents, high unemployment rates, living in social housing) and information about other health behaviours (e.g. parental smoking, low breastfeeding rates, TV watching and lack of opportunities to play outdoors for children) suggest they may be doubly disadvantaged in relation to both their living environment and their health. In this context it is gratifying that the overall response rate for the study was very high (81%) for a disadvantaged population, thus reducing the potential for respondent bias.

Participants were divided according to better/poorer dietary quality on the basis of current guidelines. No participant's child had a balanced diet according to recommendations based on the 'EatWell plate'[26] however, these recommendations may be difficult for parents of 2-3 year old children to meet. We therefore constructed more (BOGH1) and less conservative (BOGH2) criteria to represent dietary quality. Using BOGH1 criteria we showed that those with poorer diets had breakfast significantly fewer times per week, and had significantly fewer 'proper sit-down meals' than those with better quality diets. In terms of recommended frequency of behaviours, they were also less likely to agree they had a sit-down meal more than once a week, although differences were not significant for the other recommended behaviours using these criteria. However group sizes were very unequal, suggesting the sample may have been under-powered to find a significant effect. This was borne out when we used BOGH2 criteria, since these data showed behaviour scores (using both direct and indirect measures) were all significantly lower for those with a poorer quality diet.

There may be a social desirability bias operating in terms of reporting behaviours in this context (e.g. participants may know that providing breakfast is seen as socially desirable), or our single measures of self-reported behaviour may have been insufficient. However, other studies have shown similar relationships between eating behaviours and dietary quality. For example Nicklas et al. [38] reported that meal frequency and snacking were related to poorer diets, whereas consuming breakfast, eating as a family and consuming regular meals were associated with better dietary health, although their study focused on nutrients consumed rather than the broader psychosocial benefits.

The current study was novel in using the TPB to understand maternal feeding behaviour. Both direct and indirectly measured frequencies of the three behaviours were predicted by intentions and perceived control, with all models being robust and highly significant. Most studies investigating predictors of dietary quality have focused on what people eat rather than the psychosocial factors investigated here. Although it is clearly important to encourage the eating of healthy foods, motivations for family eating behaviours may be equally or more important mediators of diet in people from disadvantaged social backgrounds [3,39]. Findings confirmed the utility of this approach. For both direct and indirect measures of behavioural outcomes, effect sizes were small to medium in predicting variance in providing breakfast (19%, 49%) and medium to large for cooking from scratch (66%, 44%) and having a proper sit-down meal (62%, 74%). These figures are larger than anticipated from previous reviews of the TPB which accounted for only 16% of variance in healthy eating behaviours overall [40] reflecting the importance of cognitions for the behaviours we studied.

It is interesting to compare the slightly different pattern of responses to the two behavioural outcome variables. Actual frequency of behaviours was a more direct method of assessment, but it is important to consider that whilst it may be possible to provide breakfast every morning, many modern parents would find it difficult to cook from scratch every day. Providing family mealtimes every day of the week may be difficult when family members work irregular hours, or have other leisure activities or commitments, particularly when pre-prepared foods are widely available. Thus we used an indirect measure of adherence to 'recommended' frequency of these behaviours.

Effect sizes were also large in explaining intentions (57% for providing breakfast, 65% for cooking from scratch and 64% for having a sit down meal), comparing very favourably with the 34% of variance in intention predicted in a recent meta-analysis [40]. Attitudes and subjective norms were fairly consistently related to intentions although experiential attitudes (enjoyment) and descriptive norms were not significant predictors of providing breakfast.

It was expected that maternal perceived control over behaviour would be an important contributor to both intentions and behaviour, particularly since the children were very young. Other studies have shown clear positive associations between parental control and healthy eating in similar age groups [41]. Both self-efficacy and controllability were related to intentions in this study. However these variables were not consistently significant predictors of behaviours. Interpretation of these findings is difficult without more in-depth questioning of participants as to their understanding of perceived behavioural control, but it may be that these differences relate to the characteristics of each behaviour. For example preparing breakfast for young children tends not to require food preparation skills, but may be influenced by the demands of other children in the household or external timetables requiring organisational skills and making it less controllable. Self-efficacy may be more closely related to perceived ability or confidence in managing family demands, rather than mastery of specific skills. Preparing breakfast also was seen as the most socially desirable of the three behaviours, perhaps because this behaviour is familiar and receives more media attention. There was less normative pressure to cook from scratch or to provide a sit-down meal, suggesting that making these behaviours more socially desirable may help to increase frequency. Positive attitudes were statistically significant predictors of intention to carry out all three behaviours. It is important for health educators to ensure that mothers have clear positive information about benefits of these behaviours for their own and their child's well-being.

## Limitations of the Study

There are several factors that may have biased responses to the questions in this survey. All of the behaviours were measured using self-reported single items and ratings may have reflected social desirability in responding. This was part of a larger-scale dietary survey and we were constrained by the overall length of the questionnaire. The TPB format is limited by requiring fixed choice questions with standard wording, which do not allow for elaboration of contextual data, and some of the constructs (e.g. controllability) may be less meaningful for certain behaviours. The current sample included many single-parents or households with a single adult which would have made it seem difficult or unnecessary to provide a 'sit-down' meal. A large proportion of children in the study regularly attended nursery, and several of these received a mid-morning snack, making it seem less important to provide breakfast at home. Because our definition of cooking from scratch was broad, the difference between this behaviour and cooking other pre-prepared food may have seemed unclear. However, although using fixed choice format, the questions were presented via a semi-structured interview to participants by a researcher using a laptop in a home setting for this study. This allowed participants to discuss or seek further information if required to resolve ambiguity.

Some of our analyses may have been underpowered, and the results involved multiple statistical testing, which may have inflated our Type 1 error rate. A further limitation is the cross-sectional nature of this study which doesn't allow us to determine causal relationships - for example between dietary quality and behaviour. Further research is required to determine the processes by which behavioural cognitions are related to behaviour and behaviour to dietary quality. Our study suggests that health educators should focus on the importance of maternal feeding behaviours as well as dietary information such as about eating 'five a day', or low saturated fat diets. This study focused on mothers, and we know that other care providers such as fathers and grandparents have an important role in determining feeding behaviours. Further research could investigate the importance of family context and relationships. Nevertheless it is important to acknowledge the key role of mothers of young children in areas of social disadvantage in determining their child's health, and to support their attempts to develop skills and confidence in their parental role via enactment of healthy feeding behaviours.

## Competing interests

The authors declare that they have no competing interests.

## Authors' contributions

IKC, LI, VS, KGP, WW contributed to the conception and design of the study and acquisition of funding. PS assisted with study design and facilitated recruitment of participants. KK was responsible for questionnaire design, recruitment strategy, data collection and some analysis. VS carried out the analysis and prepared a draft of the manuscript, which was critically reviewed by IKC, LI, WW, KK and KGP. All authors read and approved the final manuscript.
